# A New Model of Master of Philosophy in Physiological Sciences

**DOI:** 10.12669/pjms.325.11481

**Published:** 2016

**Authors:** HR Ahmad, FM Arain, NA Khan

**Affiliations:** 1 HR Ahmad, Department of Biological and Biomedical Sciences, The Aga Khan University, Karachi, Pakistan; 2 FM Arain, Department of Biological and Biomedical Sciences, The Aga Khan University, Karachi, Pakistan; 3 NA Khan, Department of Biological Sciences, Sunway University, Malaysia

**Keywords:** MPhil, Faculty-development, Research-based education

## Abstract

The objectives of Master of Philosophy (MPhil) in Physiological Sciences are: 1) to describe the new ways in which anatomy, biochemistry and physiology on one hand, and microbiology, pathology and pharmacology on other hand meet their functional requirements through multidisciplinary integrated concepts; 2) to elucidate relationships between cell biology, molecular biology and molecular genetics by connecting dots of how cell functions are driven by molecules and being controlled by genes. This forms the basis of cell, molecular and genetics [CMG] module upon which 7 multidisciplinary modules of Physiological Sciences follow; 3) these 24 credit hours provide the physiological basis for PhD studies as well as faculty development to enhance learning abilities of medical student; 4) the modules constitute Cardio- Respiratory Physiological Sciences, GI and Renal Physiological Sciences, Neurosciences, Endo-Reproductive Physiological Sciences.; 5) it has integrated microbiology, pathology and pharmacology in a unique way through CMG of microbes leading to associated pathology and mechanisms of prescribed drugs; 6) it has additional synopsis and thesis friendly course work leading to comprehensive examinations; 7) the year two deals with research work of 6 credit hours leading to defense of thesis; 8) The MPhil in Physiological Sciences is fundamentally different from what is being offered elsewhere. It prepares and offers a good spring board to dovetail PhD studies as well as faculty and institutional development.

This is the first study that deals with innovative programmes in research, learning and education in the field of physiological sciences. This broad-based MPhil would make its recipients competent, critical, confident and productive learner. This is a completely unique design of a curriculum that has no comparable examples elsewhere. Our mission is to educate graduate students in the field of Physiological Sciences such that they have a complete grasp over the broad-based integrated concepts of basic health sciences. Upon completion of their education, the students will be able to use the duality of imagination and skepticism. Hence, the students will contribute to their fields by unfolding their creative energy.

## INTRODUCTION

The goal is to identify and nurture graduate students with the talent and passion for research through MPhil in physiological sciences. They would learn to sail a boat of research based education fueled by the spirit of scientific curiosity in pursuit of knowledge.

This MPhil Programme is primarily needed for sustenance of an institution’s ability, capacity and resources. Since there is dearth and need of faculty for undergraduate teaching/learning and research of basic sciences in the inflated number of medical schools of a country, this may fulfill the faculty demand as well as secure grants for research to promote faculty development at the institutional level.

This MPhil Programme will lead to PhD studies in order to establish the loop of pre-doctorate, doctorate and post-doctorate fellows to derive the generation cycle of an institution. The objectives of the academic plan would be to establish research based education, to integrate the desired attributes of knowledge, skills and attitude as outcomes and to welcome meritorious applicants from wide range of disciplines.

The academic plan will focus development of breadth and depth of knowledge in the form of multidisciplinary integrated concepts, self-directed learning, inquiry, critical thinking and expression of thought to strengthen the model of research based education through a horizontal enabling environment. The assessment and certification will include statements of what will be assessed and how will validity be determined. The assessment of quality, originality and significance of course and research work and the relation between assessment and learning will be monitored. The multidisciplinary postgraduate faculty will primarily drive this program. Additionally, PhD students will take a portion of undergraduate teaching as teaching assistants. The academic programme will start with a reasonable assurance of continuity, which will be provided by institutions. This will be supplemented by both internal and external research grants.

### Programme Design

The program consists of course work and research as summarized in table 1 and 2. The former is aimed to strengthen the multidisciplinary integrated concepts of physiological sciences as a solid foundation for developing research mentors and educators. This will include research friendly courses to aid efforts in preparing and defending a synopsis before the reviewers and faculty. The experience shows that a well prepared and well-designed synopsis is half done. The next step would be research work after passing the comprehensive examination and the defense of synopsis. This action would be with a team of research mentor and mentee supported by institutional faculty and facility. This process would lead finally to a thesis as a tool of discovery of new facts or fresh interpretation. A successful defense of thesis would show the candidates’ ability of critical thinking and reasoning. Based on this original performance of *research based education*, the degree of MPhil in Physiological Sciences would be conferred. This, in turn, would enable candidates to be invited for PhD studies on merit of talent and passion for research.

**Table-I:** Details of multidisciplinary integrated concepts: modules are designed to cover all major fields of physiological sciences.

*001-CMG Module*
Cellular and Molecular Physiological Sciences
1.1. Cell structure and Function
1.2. Signal Transduction
1.3. Membrane Transport
1.4. Cell Excitability
*002-CVR Module*
Cardiovascular and Respiratory Sciences
2.1. Cardiac Mechanics
2.2. Cardiac Cycle
2.3. Cardiac Rhythm
2.4. Haemodynamics
2.5. CVS Control Systems
2.6. Pulmonary Mechanics
2.7. Gas Exchange
2.8. Gas Transport
2.9. Respiratory Control Systems
*003-RGI Module*
Renal and GI Sciences
3.1. Perfusion and Filtration
3.2. Reabsorption and Secretion
3.3. Renal Control Systems
3.4. Peristalsis
3.5. Digestion and Absorption
3.6. GI Control Systems
*004-NS Module*
Neurosciences
4.1. Neuronal Circuits
4.2. Sensory System
4.3. Motor System
4.4. Special Senses
4.5. Autonomic Nervous System
*005-ERS Module*
Endocrine and Reproduction Sciences
5.1. Synthesis, Storage and Secretion
5.2. Functions of Hormones
5.3. Hormonal Control Systems
5.4. Gametogenesis
5.5. Regulation of Gonadal Activity
*006-Hematology Module*
Hematological Science
6.1. Blood volume and hematocrit
6.2. Blood plasma
6.3. RBCs and haemoglobin
6.4. WBCs
6.5. Immunity
6.6. Blood groups
6.7. Platelets
*007-MPP Module*
Microbiological, Pharmacological and Pathological Sciences
7.1. CMG Concepts of Microbes
7.2. Associated Pathology
7.3. Associated Pharmacology
*008-Additional Courses*
008.1. Research Methodology and Biostatistics
008.2. Research Elective
008.3. Creative Thinking
008.4. Journal Article Reading/Writing
008.5. Assignments
008.6. Faculty-Student Seminars
008.7. Integrated concepts of Mathematics Physics and Chemistry
008.8. Integration of Arts and Sciences

**Table-II:** Duration and credit hours of course contents. A summary of how different modules could be chosen according to the required design of a curriculum.

*Module Course*	*Duration*	*Credit hours (CH)*
001-Molecular and Cellular Physiological Sciences	05 weeks	5 CH
002-Cardiovascular and Respiratory System	05 weeks	5 CH
003-Renal and GI Sciences	03 weeks	3 CH
004-Endocrine and Reproductive Sciences	03 weeks	3 CH
005-Neuroscience	05 weeks	5 CH
006-Hematology	02 weeks	2 CH
007-Microbiological, Pathological and Pharmacological Sciences	05 weeks	5 CH
*008: Additional Courses*		
Research methodology and Statistics	36 weeks	0.5 CH
Research electives	36 weeks	1 CH
Creative and Critical Thinking	36 weeks	0.5 CH
Journal Article Reading and writing Course	36 weeks	0.5 CH
Assignments	36 weeks	3 CH
Faculty and Research Seminars	36 weeks	0.5 CH
Concepts of Mathematics, Physics and Chemistry	36 weeks	1 CH
Integration of Arts and Science	36 weeks	2 CH
**Total Duration:**	36 weeks	37 hours
*Comprehensive Exam*		
009: Thesis Research	36 weeks	18 CH

### Modules that will be part of the curriculum are

A) ***Research Methodology***: The three research dimensions are: 1. Design; 2. Setting; 3. Data bank. The research design deals with experiments, correlational and descriptive studies. The setting includes laboratory, ward and field. The information from self-report interview and observation flows into the data bank for analysis and summary. Since each of these dimensions can vary from each other, twelve different studies can be generated. When we design an experiment based on our observations, we are usually curious to know how an experiment can prove the existence of a cause-effect relation between two variables. The cycle of science consists of observations – experiments – data analysis – hypothesis to explain data – theory – law. Theory is a conceptual model to explain existing data with a hypothesis which is at the same time has a predictive value. This is a non-hypothesis based research in contrast to hypothesis based research. Both research pathways are being practiced.
B) ***Research Design***: In an *experiment*, the researcher can investigate the *cause-effect relationship* by manipulating the independent variable and look for the corresponding effect on the dependent variable. However, known confounding variables should be kept close to constant. In a *correlational study*, a systematic relationship between two variables is investigated. *Descriptive studies* are designed to characterize and record what is observed.
C) ***Research Settings***: The control over the variable is best achieved in the *laboratory settings* while taking into consideration of unfamiliar and artificial environment. *Bed-side and field* offer “real life” settings but with less control on the variables of research study.
D) ***Data Bank***: *Self-reporting* deals with subject’s observations on what they rate and describe using the tools of questionnaires and interviews. The data from experimental biology with a sensitive and specific recording system and a sound analysis enable researchers to compare how the data can be explained by hypothesis. The influence on the observed effects of a study by chance or not-by chance can be processed by statistical methods.
E) ***Statistical Methods***: The statistical procedures are primarily of two types namely *descriptive and inferential* *statistics*. The descriptive statistics deals with how to summarize sets of numerical data using median, means, standard deviation, standard error of mean, sample size and correlation coefficient. The inferential statistics can resolve the contradiction whether or not the observed results are due to chance caused by uncontrollable random variables or not-by chance predicting repeatable effect using the laws of probability. The statistical significance depends on the size of the effect, the number of observations, and the variability from the central tendency within each group. The key words for the descriptive statistics are data summary, central tendency, standard deviation and correlation coefficient. Inferential statistics tells us whether the results are both real and repeatable or merely due to chance. Thus, statistical significance should mean the study is real and reproducible.
F) ***Research Elective***: After successful completion of the comprehensive examination, the student would acquire a new status known as a candidate MPhil. Now the candidate would enter the phase of research elective leading to a synopsis to be defended. The three months elective period would enable the candidate to familiarize with the methodology and literature survey being used in the research work. This hands-on experience and the literature study are an essential motor of a thesis. At the same time the candidate can start a pilot project on a topic being agreed between the mentor and the mentee. This elective should equip the candidate with the know-how to collect data, organize, choose methods of analysis, formulate hypothesis to explain the data. The assessment will be done during the period of synopsis defense before the peers. The research elective could expand over wet laboratory hands-on experience, bio-informatics tools, ward and field settings dealing thus with various streams of disciplines.
G) ***Creative and Critical Thinking Course***: This course spans over four topics in series. These are: 1. Awareness; 2. Thinking; 3. Critical Thinking and 4. Communication. This course aims to unfold the creative energy of students through an enabling environment of exchange of notes. It would instill in students the capacity of conscientious thinking to enable them not only to welcome but also celebrate differences. It is anticipated that this course will make students more resilient to better respond to stress-and-crisis-situations and provide them the ability of critical thinking to solve problems.
H) ***Journal Article Reading/Writing Course***: Journal articles will be critically read together with a team of students till the article has been thoroughly discussed and debated. It primarily serves to empower them with the skills of analyzing scientific manuscripts. If articles are well understood and gaps in the literature are identified, this capacity of comprehension aids in writing synopsis, thesis and publications. It enables them the art of staging debate through good listening and critical reasoning. Four to six articles will be read over a period of three months duration. Emphasis will be given to writing skills after reading the articles. This activity will be evidenced by how good they can write summaries of critically read articles. It is anticipated that this mode of in-depth analysis of scientific matter will reveal insightfulness and enlightenment in students. This course together with the laboratory electives will be a powerful tool of research based education.
I) ***Assignments***: This is a form of an informal assessment to discover ‘candidates’ independent scholarship activity. They will work on various assignments both in the theoretical and practical domains. The example of a theoretical work could be writing a mini-review article on a topic of choice. Experimental work may include how to do an accuracy study of an instrument. They will also be given take-home assignments from course and out of course work. This should initiate discussion and debate on matters of mutual interest between faculty and students. This will enable them to work independently.
J) ***Faculty Research Seminar***: The state of the art lectures by faculty will offer students opportunity to be exposed to the research forum. Active participation of students will be observed in context of how good they are in asking critical questions and stage debate with the speaker. This would also enable a student to choose one of the speakers as a mentor to pursue the research elective. Students will be required to submit a summary after each faculty research seminar which will be assessed.
K) ***Integrated Concepts of Mathematics, Physics and Chemistry***: This course on the integrated concepts of mathematics, physics and chemistry has been designed to refresh and instill the basic critical thinking to enable research based education. Therefore, these three pillars of science need to be strengthened. This is a team-taught course where the lecturers from three domains will form a panel to discuss and debate basic concepts as a foundation stone for understanding physiological sciences. This course would enlighten how physics can become biophysics. This course will give us clue how elements from the periodic table evolved to make a cell. Since most of the synopses of science is expressed in mathematical formulae like a half inch formula of Einstein E = mc^2^, the application of mathematical concepts would enable them to distill their research findings. This course would wing students with ability and capacity of critical thinking to appreciate life through the lenses of the laws of nature.
L) ***Integration of Arts and Sciences***: Since imagination and skepticism are the fundamental tools of science, this integration would enable the students to stimulate imagination through the concepts from disciplines of arts. Imagination opens new horizons while skepticism enables us to differentiate fancy from facts. This would mean how to deal with the dearest delusion against bitter fact. This integration would enable us how to loop philosophy with science, where in one domain questions are generated and refined while the other domain answers them. Thus a national endowment for arts and sciences would provide the required ideal environment for science to drive research-based education. This would lead to the conception of a humanistic society with a sense of diversity, vitality and excitement of enlightenment. Such an enabling environment would provide a field out of which “renaissance” citizens could be born like a Higgs-field interacting with particles to enable a diversity of atoms leading the journey to consciousness.

## ASSESSMENT

Finally the assessment needs to be well designed and provide a holistic overview of the student’s progress. The question to be raised is how assessment and certification of courses will be assessed and how validity is determined in context of quality, originality and significance for both coursework and research work. Of note is the question of relevance how to relate mode of assessment with the learning curves driven by the interaction of student and multidisciplinary faculty. The outcome of assessment should be to discover whether or not the elements of curiosity and thinking are instilled through courses and evidenced by outcome of assessment. This would form the basis of competencies at graduation. This in turn would show how good the MPhil studies could become a spring board for PhD studies. The components of assessment will include the following:

- 30% marks for extended integrated assignments assessing the understanding and application of concepts
- 30% marks for experimentation based problem solving questions
- 20% marks for integrated extended matching questions with emphasis on understanding and comprehension
- 20% critical appraisal of journal article publications

The quality assurance would require the following monitoring steps where quality is understood to be prerequisite for fitness of purpose.

1: Support for learning includes
  - Learning objectives, laboratory and infrastructure
  - Multidisciplinary preparation of knowledge and skills being driven by faculty-student loop.
  - Portfolio assessment is aligned with active learning approach.
2: Certification demands integrity of processes and security of papers.
3: Public accountability will look for reference assessment procedures, international equivalence and social responsibility. The integrity and reliability of the marking process would achieve to prevent impersonation, reducing assessment error to < 2% and generate diagnostic feedback.

## DISCUSSION

This manuscript offers a new way of thinking how to develop MPhil studies based both on breadth and depth of knowledge in the form of integrated multidisciplinary concepts to use the loop of imagination (brain) and experimentation (hands). The competencies at graduation will include: thinker, problem solver, scholar with integrity of professionalism and honesty. The content, strategies and assessment are designed to unfold competencies of students by providing them a learning friendly enabling environment. A successful MPhil should serve as a good spring board for PhD studies.

MPhil studies include a year of coursework of 7 modules and thesis friendly courses and a year of research work. The invited candidates for MPhil will start the course with the CMG module. This course will deal with the structure and function of a cell from the point of view of how molecules made a cell and how then cells made us. A three compartment model would lay the foundation of cell biology: genes – proteins - functions to understand the behavior of a cell. In this way, cell biology, molecular biology and genetics will be integrated. This will show how genes encode proteins to ensure a given function of a cell. The knowledge of genetic encyclopedia would elucidate how this gene library functions to provide precoded information for a daily life activity. Thus, this CMG module would function as a foundation stone to understand each organ system of a body.

The CVR module deals with how anatomy, biochemistry and physiology are integrated to elucidate the underlying mechanisms of cardiovascular and respiratory sciences. Likewise follows the modules of renal, gastrointestinal, neurosciences and endocrine and reproduction. Of note is the fact that the neuroscience module will be integrated on the basis of three loops: 1) body-brain, 2) brain-body and 3) brain-brain using CMG module as a base.

How to integrate microbiology, pathology and pharmacology was a challenge. However, multiple brainstorming resulted in an ingenious solution. This module deals with CMG of four microbes: 1) virus, 2) bacterium, 3) amoeba and 4) fungus with increasing trend of bits of information to survive and reproduce. Once the CMG of four microbes are elucidated with their mechanisms of lifestyle, triggers will be identified that change their characteristics toward pathology of a host. Pathology caused by each microbe will then be studied. This will be followed by how the pathology could be reversed by the use of molecular immunology- and drug-based therapy in context of molecular mechanisms of action.

The following additional courses will enable students to link themselves to the enlightenment of science with aim to unfold their creative energy. The dialects of imagination and experimentation will be instilled in them through the activation of brain – hand loops, typically known as psychomotor skills for research. Of note is the fact that this proposed framework of MPhil can be applied where integration of various disciplines and research based education are sought. This may offer a unique opportunity to transform an existing training system into education. Training is good but education is better. The training generates followers while education liberates fellows to be detectives of laws of nature to decode the encoded mechanisms of cause and effect phenomena.

## CONCLUSION

This manuscript on MPhil in physiological sciences serves as prerequisites for understanding multidisciplinary integrated concepts of basic medical sciences. This would enable us to understand the problems to derive solutions. This two year broad-based graduate curriculum is covered in two phases. Phase one deals with the multidisciplinary integrated concepts of domains of cell, molecular biology, genetics, anatomy, biochemistry and physiology on one hand and microbiology, pathology and pharmacology on the other hand. Phase two deals with the synopsis and thesis friendly courses leading to active independent research work. This two year scholarly activity would reveal the interaction of individual student’s experiences, skills and motivation with the opportunities and enabling environment of an institution. It means each candidate has to look for what pre-knowledge she/he is bringing to the institution in order to reap the fruits of new knowledge. The uniqueness lies in the fact that this model is different from the discipline based MPhil studies being practiced elsewhere. This new model in this way prepares students ideally for PhD studies. It enables one to proceed on a solid foundation of a broad based coursework. It is supplemented by an MPhil research project to dovetail PhD studies. We may conclude that this double engine model of MPhil leading to PhD provides an excellent opportunity for students and faculty to enlighten a society by implanting a research culture of arts and sciences to serve humanity.
